# Dynamical Calibration of a Standard Sensor-Based Experimental Test Apparatus for Accurate Material Model Parametric Identification

**DOI:** 10.3390/s25071982

**Published:** 2025-03-22

**Authors:** Giuseppe Catania, Stefano Amadori

**Affiliations:** 1 Department of Industrial Engineering (DIN), Ciri-Mam, University of Bologna, Viale Risorgimento 2, 40136 Bologna, Italy; giuseppe.catania@unibo.it; 2 Ciri-Mam, University of Bologna, Viale Risorgimento 2, 40136 Bologna, Italy

**Keywords:** test calibration, dynamical model identification, nondestructive experimental measurements

## Abstract

A signal processing-based procedure is proposed for calibrating an experimental sensor-based test system used to identify material model parameters. A standard dynamic mechanical analyzer (DMA) sensorized test apparatus is considered, enabling the measurement of dynamic excitation and displacement response in a specimen under flexural conditions. To account for the dynamic contributions of the system frame and fixtures to the measured response, a novel calibration procedure is introduced, mainly differing from the techniques used in standard test applications. A multi-degree-of-freedom dynamic model of the instrument frame, coupled with the beam specimen under test, is considered, and a frame identification procedure is described. The proposed procedure requires measurements from at least three beam specimens made of a known material but with different geometries. It is shown that an accurate frame model can be identified using an algebraic numerical technique. It is shown that the accuracy of the material model identification can be improved by applying the proposed calibration technique. Some experimental application examples are presented and discussed.

## 1. Introduction

The model parameters of a homogeneous isotropic viscoelastic material can be identified through input–output dynamic measurements on a specimen made of the material under study. Typical mechanical test configurations include free or forced excitation response measurements in uniaxial tension, compression, flexure, shear, torsion [1,2,3,4], or more general multiaxial stress configurations [5,6]. A sensorized test machine designed for this purpose generally consists of a rigid frame, test fixtures that allow the mounting of various specimens, a sensorized subsystem for measuring input excitation and output response, and a computer-based subsystem for analog data acquisition, digital conversion, and signal processing. Input–output frequency response functions (FRFs) are typically estimated and can be used to identify any of the two independent model parameters related to an isotropic homogeneous material, such as the *E*(*ω*) tension–compression and the *G*(*ω*) shear complex moduli [7,8], where *ω* is the angular frequency. DMA systems are an established experimental tool [9,10,11,12,13] to be used for the identification of the dynamical properties of a wide range of materials. Assuming the measured system’s input–output FRF linearly varies with the material parameter being estimated, identification can be obtained by averaging multiple measurements [7].

It is important to emphasize that dynamical measurements in the low to medium frequency range are expected to be significantly influenced by the contribution of the test system frame, the coupling fixtures, the moving mass inertia, the temperature range, and the instrument sensors used to obtain such measurements [7,14,15]. It is thus always necessary to calibrate the system to take into account these contributions. As a matter of fact, Menard [7] highlighted the importance of calibrating the DMA instruments, as when testing stiff samples, instrument deformation could potentially exceed sample deformation, leading to inaccurate results. Duncan [16] discussed the contribution of the clamping fixture subsystem on material modulus estimates derived from force and displacement measurements of various specimen geometries.

The identification of material parameters from dynamical input–output measurements requires an accurate test system model being known in advance, such model including the contribution of the specimen model and of the coupled frame subsystem model. The choice of the mechanical model for the material specimen under test is generally an easy task, since effective mechanical models are known from literature and can be easily adopted, provided that the specimen geometry respects some requirements. The same is not true with respect to the model of the other mechanical subsystems coupled to the specimen under test. Nevertheless, the instrument frame contribution is sometimes neglected in some applications [6,17,18,19], i.e., ideal boundary conditions are assumed, and the material identification results are generally expected to be strongly affected by this assumption.

The frame contribution is sometimes modeled using a discrete lumped parameter subsystem, typically represented by a single elastic spring, with its parameters identified by means of a preliminary calibration measurement procedure [7,12,17]. The literature and known technical applications describe other approaches to model the instrument frame contribution by means of continuous or n-degrees of freedom (dof) discrete structural or modal analytical models [14,20]. Experimental models were proposed [15,21], primarily relying on in situ measured frequency response function (FRF) estimates at specific input–output locations of the measurement machine. Each approach has its advantages and limitations. Ideal assumptions are only applicable when measuring highly compliant specimens in a very low frequency range. In contrast, n-dof frame subsystems require complex modeling steps and several simplified assumptions. Experimental models may demand advanced measuring techniques, can be seriously affected by experimental noise, and may require measurement of the excitation and response in non-accessible system locations.

For example, Fisher et al. [22] proposed a modeling of the frame contribution using a single elastic lumped parameter, to be identified in a quasi-ideal three-point bending experimental set-up. However, this set-up was formally different from the actual system frame configuration used in the material identification experiments, making the proposed calibration not consistent. Kalidindi et al. [23] experimentally determined nonlinear frame compliance as a function of the applied load to calibrate the static material modulus identification results, an approach that was also adopted by Davidson et al. [24]. However, no attempt was made to account for the dependence on the measurement excitation frequency, and the identified model parameters are assumed to be valid only in a low, quasi-static frequency range.

Standard calibration procedures for identifying the frequency-dependent storage and loss modulus—i.e., the real and imaginary components of the estimated modulus—to be used with both commercial and custom built dynamic mechanical testing systems are known and were proposed by ASTM [25,26]. ASTM procedures rely on dynamic force and displacement measurements performed on specimens with known geometry and material properties, making it possible to experimentally find a complex coefficient to calibrate the identified material parameters. This calibration procedure is assumed to be valid for a wide range of materials tested at different frequencies. But this appears to be a critical assumption, since the ASTM procedure could only be applied to identify material properties within a limited frequency range. In the context of uniaxial dynamic nanoindentation measurements, White et al. [27] proposed a calibration of the test measurements by experimentally identifying the instrument sensor’s moving mass and the elastic and viscous parameters of a lumped suspension put in parallel with the specimen under test. Nevertheless, it is expected that the constant parameters modeling the suspension behavior strongly vary with respect to frequency, making this calibration approach consistent only in a very limited frequency range.

In the context of *E*(*ω*) and *G*(*ω*) estimates, the dynamic contribution of the instrument frame is expected to vary significantly with the measurement excitation frequency, even within the low to medium frequency range. In a previous study, the authors proposed a calibration procedure based on a multi-dof frame model identification procedure. This method requires only input/output measurements, which can be obtained from a commercially available DMA instrument system [28], using specimens whose model behavior is assumed to be known in advance. The procedure was applied in the context of standard flexural excitation measurements of slender beam specimens aimed at the identification of the single *E*(ω) parameter. The *E*(ω) estimate was derived from input–output measured FRFs on a specimen made of the material under test, filtered from the beam specimen ideal mathematical model and from the previously identified dynamic models of the frame and of the sensorized measuring subsystem. The procedure was based on the Euler–Bernoulli beam theory to model the beam specimens used for the instrument frame calibration. In principle, the same approach could be applied to identify *G*(*ω*) through uniaxial shear measurements performed on different specimens, all made from the same material. However, the accuracy of the identified material model is influenced by the use of different specimens and experimental set-ups. As previously found by Fisher et al. [22], the same flexural experimental set-up can be used to identify both *E*(ω) and *G*(ω), with the caveat that a high measurement accuracy is needed, since the shear stress contribution is significantly lower than the axial stress contribution to the beam response. To reduce model error noise, the contribution of the experimental system frame to the specimen’s transverse deflection must be accurately evaluated to increase the accuracy of both *E*(ω) [28] and especially *G*(*ω*) identification.

This work describes a novel calibration technique for the calibration of a DMA measuring system, with the aim of overcoming the limitations shown by existing techniques. The previously proposed calibration procedure is here extended by modeling the test beam specimens used in the frame calibration procedure with Timoshenko beam theory [29]. This allows a relaxing of the slender beam assumption so that deep beams can be accounted for as well. In this work, a procedure is introduced for identifying a multi-dof instrument frame model, which is then coupled with the model of the material test specimen under test. The effectiveness of this approach is evaluated by comparing the results obtained using the proposed frame calibration procedure with the results obtained with the previously cited frame calibration algorithm [28].

## 2. Experimental System Model

**Figure 1:**
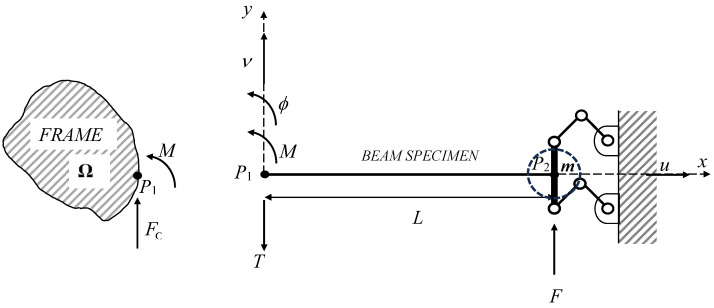
Experimental system model.

The model of the experimental set-up is shown in Figure 1. *L* is the beam length, while *x*, *y* refer to a cartesian coordinate system centered with respect to the left beam side and *x* is associated with the beam axis. A *ξ* = *x*/*L* normalized coordinate is introduced, and *u*, ν, *ϕ* are the *x*,*y* displacement and rotation components of the beam response, respectively. *F* is the excitation force and *M* and *T* are the bending moment and shear resultants of the beam cross-sectional distributed actions. A uniform homogeneous beam specimen with a rectangular cross section is harmonically excited at fixed discrete frequency values in correspondence with the (*ξ* = 1) beam end. Both the *F* force and *ν* transverse displacement complex response at the (*ξ* = 1) beam end, accounting for both response amplitude and time delay with respect to the excitation, are estimated from measurements. The other (*ξ* = 0) beam end is coupled to the instrument frame, which is modeled using a 2 × 2 **Ω**(ω) symmetric transfer function matrix in the frequency domain:

(1) $$\left[\begin{array}{c} v\left(\xi =0\right)\\ \phi \left(\xi =0\right) \end{array}\right]=\Omega \left(\omega \right)\cdot \left[\begin{array}{c} F_{\Omega }\\ M_{\Omega } \end{array}\right]=\left[\begin{array}{cc} \Omega_{11}\left(\omega \right) & \Omega_{12}\left(\omega \right)\\ \Omega_{12}\left(\omega \right) & \Omega_{22}\left(\omega \right) \end{array}\right]\cdot \left[\begin{array}{c} F_{\Omega }\\ M_{\Omega } \end{array}\right].$$

By imposing the equilibrium condition at *ξ* = 0, *F*_Ω_ = −*T*(*ξ* = 0) and *M*_Ω_ = *M*(*ξ* = 0) hold. The (*ξ* = 1) beam end is coupled to the instrument mobile measuring subsystem under double pendulum boundary conditions (*ϕ*(*ξ* = 1) = 0). The inertial contribution of the instrument mobile measuring subsystem is modeled by the *m* mass lumped at (*ξ* = 1), as illustrated in Figure 1. This set-up leads to four boundary conditions:

(2) $$\left\{\begin{array}{l} \left[\begin{array}{c} \nu \left(\xi =0\right)\\ \phi \left(\xi =0\right) \end{array}\right]=\Omega \cdot \left[\begin{array}{c} F_{\Omega }\\ M_{\Omega } \end{array}\right]=\Omega \cdot \left[\begin{array}{c} -T\left(\xi =0\right)\\ M\left(\xi =0\right) \end{array}\right]\\ T\left(\xi =1\right)=-F\\ \phi \left(\xi =1\right)=0 \end{array}\right..$$

It must be outlined that the conditions shown in Equation (3) are also assumed to hold for a rigid frame within the low to medium frequency experimental range:

(3) Re(Ωω)≥0, Im(Ωω)≤0.

By imposing the Equation (2) beam boundary conditions, the analytical expression of the beam FRF at *ξ* = 1 can be derived (see Appendix A for details):

(4) $$H\left(\omega ,v\Omega \left(\omega \right)\right)=\frac{v\left(\omega ,\xi =1\right)}{F}=d\left(\omega ,E\left(\omega \right),G\left(\omega \right),1\right)\cdot D^{-1}\left(\omega ,E\left(\omega \right),G\left(\omega \right),v\Omega \left(\omega \right)\right)\cdot N$$

where **vΩ** = {Ω_11_, Ω_21_, Ω_22_}^T^, with ( )^T^ denoting the transpose operator. Vector **d**(*ω*,*ξ*) is detailed in Equation (A10), while **N**, **D** are introduced in Equation (A16). To identify **vΩ**(*ω*), a set of *h_s_*(*ω_i_*) = (*ν*/*F)*(*ω_i_*), *s* = 1...*N_m_*, *i* = 1…*Nω* FRF measurements from *N_m_* reference specimens are considered here. The reference specimen geometry and *E*(*ω*) and *G*(*ω*) reference material parameters are assumed to be known in advance, e.g., by employing reference specimens made of harmonic steel material.

To identify **vΩ**, *N_m_* ≥ 3 measurements at each *ω_i_* value are needed since the **vΩ**(*ω*) unknown vector contains three independent elements. Equation (4) can be used to obtain a set of *N_m_* equations related to the error between measured and estimated FRF values.

At each *ω_i_* value, the differences between the *h_s_*(*ω_i_*) measurements and the *H_s_*(*ω_i_*) theoretical values (from Equation (4)) form the elements of the **e** error vector:

(5) $$e\left(\omega_{i},v\Omega \left(\omega_{i}\right)\right)=\left\{\begin{array}{c} e_{1}\\ \ldots \\ e_{m} \end{array}\right\}=\left\{\begin{array}{l} h_{1}\left(\omega_{i}\right)-{\left(d\left(\omega_{i},1\right)\cdot D^{-1}\left(\omega_{i},v\Omega \left(\omega_{i}\right)\right)\cdot N\right)}_{1}\\ \ldots \\ h_{N_{m}}\left(\omega_{i}\right)-{\left(d\left(\omega_{i},1\right)\cdot D^{-1}\left(\omega_{i},v\Omega \left(\omega_{i}\right)\right)\cdot N\right)}_{N_{m}} \end{array}\right\}.$$

It must be outlined that Equation (5) is highly nonlinear with respect to the **vΩ**(*ω_i_*) unknown vector. To find the optimal **vΩ**(*ω_i_*) value, a two-step approach is adopted.

An approximate expression of Equation (4) related to the *s*-th FRF specimen is considered here (see Appendix A for details):

(6) $$H_{s}\left(\omega ,v\Omega \left(\omega \right)\right)≃d\left(\omega ,1\right)\cdot A^{-1}\left(\omega ,v\Omega \left(\omega \right)\right)\cdot B\cdot v\Omega \left(\omega \right)+d\left(\omega ,1\right)\cdot A^{-1}\left(\omega ,v\Omega \left(\omega \right)\right)\cdot C$$

where **A**, **B**, **C** are detailed in Equations (A20) and (A21).

The following result can be obtained by considering, at each *ω_i_* value, the difference between the *h_s_*(*ω_i_*) measured values and the *H_s_*(*ω_i_*) theoretical values (Equation (6)):

(7) $$\begin{array}{l} {\left(d\left(\omega_{i},1\right)\cdot A^{-1}\left(\omega_{i},v\Omega \left(\omega_{i}\right)\right)\cdot B\right)}_{s}\cdot v\Omega \left(\omega_{i}\right)≃h_{s}\left(\omega_{i}\right)-{\left(d\left(\omega_{i},1\right)\cdot A^{-1}\left(\omega_{i},v\Omega \left(\omega_{i}\right)\right)\cdot C\right)}_{s},\\ \;s=1\ldots N_{m} \end{array}$$

From Equation (7), in compact form, the following nonlinear system of *N_m_* equations in the **vΩ**(*ω_i_*) unknown vector results:

(8) $$\begin{array}{ll} v\Omega \left(\omega_{i}\right) & =\Phi \left(v\Omega \left(\omega_{i}\right)\right),\;\;\;\;\\ & \Phi \left(v\Omega \left(\omega_{i}\right)\right)=BB^{-1}\left(\omega_{i},v\Omega \left(\omega_{i}\right)\right)\cdot CC\left(\omega_{i},v\Omega \left(\omega_{i}\right)\right),\\ & CC\left(\omega_{i},v\Omega \left(\omega_{i}\right)\right)=\left[\begin{array}{c} h_{1}\left(\omega_{i}\right)-{\left(d\cdot A^{-1}\left(\omega_{i},v\Omega \left(\omega_{i}\right)\right)\cdot C\right)}_{1}\\ \ldots \\ h_{m}\left(\omega_{i}\right)-{\left(d\cdot A^{-1}\left(\omega_{i},v\Omega \left(\omega_{i}\right)\right)\cdot C\right)}_{N_{m}} \end{array}\right],\\ & BB\left(\omega_{i},v\Omega \left(\omega_{i}\right)\right)=\left[\begin{array}{c} {\left(d\left(\omega_{i},1\right)\cdot A^{-1}\left(\omega_{i},v\Omega \left(\omega_{i}\right)\right)\cdot B\right)}_{1}\\ \ldots \\ {\left(d\left(\omega_{i},1\right)\cdot A^{-1}\left(\omega_{i},v\Omega \left(\omega_{i}\right)\right)\cdot B\right)}_{N_{m}} \end{array}\right]. \end{array}$$

From Equation (8), the following iterative procedure is proposed to evaluate **vΩ**(*ω_i_*), starting from the initial value condition **vΩ**_0_(*ω*_1_) = **0** and **vΩ**_0_(*ω_i_*) = **vΩ**(*ω_i−_*_1_):

(9) $$v\Omega_{k}\left(\omega_{i}\right)=\Phi \left(v\Omega_{k-1}\left(\omega_{i}\right)\right),\;\;k=1\ldots end.$$

The end condition is:

(10) $$\begin{array}{l} v\Omega \left(\omega_{i}\right)=v\Omega_{end}\left(\omega_{i}\right):\\ \;\;\left|v\Omega_{end}\left(\omega_{i}\right)-v\Omega_{end-1}\left(\omega_{i}\right)\right|<tol_{x}\;\;OR\;\;\;\;{\tilde{e}}^{*}\left(\omega_{i},v\Omega \left(\omega_{i}\right)\right)\cdot \tilde{e}\left(\omega_{i},v\Omega \left(\omega_{i}\right)\right)<tol_{e}, \end{array}$$

where *tol_x_* and *tol_e_* are user-defined tolerance values and ( )* denotes the complex conjugate operator. $\tilde{e}$ is obtained from Equation (7):

(11) $$\tilde{e}\left(\omega_{i},v\Omega \left(\omega_{i}\right)\right)=\left\{\begin{array}{l} h_{1}\left(\omega_{i}\right)-{\left(d\left(\omega_{i},1\right)\cdot A^{-1}\left(\omega_{i},v\Omega \left(\omega_{i}\right)\right)\cdot \left(B\cdot v\Omega \left(\omega_{i}\right)+C\right)\right)}_{1}\\ \ldots \\ h_{N_{m}}\left(\omega_{i}\right)-{\left(d\left(\omega_{i},1\right)\cdot A^{-1}\left(\omega_{i},v\Omega \left(\omega_{i}\right)\right)\cdot \left(B\cdot v\Omega \left(\omega_{i}\right)+C\right)\right)}_{N_{m}} \end{array}\right\}$$

It must be outlined that if, at the *k*-th iteration step, the value of **vΩ***_k_*(*ω_i_*) obtained from Equation (9) does not satisfy Equation (3) conditions, a specialized algorithm [30] is employed. This algorithm makes it possible to obtain the least-squares solution of a linear system of equations constrained by inequalities, ensuring that the obtained **vΩ***_k_*(*ω_i_*) values remain consistent with the requirements of Equation (3).

This algorithm is found to be robust, as **Φ**(**vΩ**(*ω_i_*)) and the initial value condition typically satisfy the Banach–Caccioppoli contraction requirements [31,32] for any *ω_i_* value within a local compact domain in the proximity of the unknown solution. Consequently, only a small number of iterations are required for the algorithm to converge. Nevertheless, since convergence mainly depends on the choice of the initial value condition, a test convergence procedure, based on [32], was implemented and is discussed in Appendix B. It was observed that such a test procedure may sometimes fail at *ω* = *ω_1_*, and in such cases, a new initial condition satisfying the test condition can be automatically determined using a nonlinear optimization procedure. It was also found that the test condition generally holds if the Δ*_i_*= *ω_i_* − *ω_i_*_−1_ value is low enough.

Since the iterative procedure described in Equation (9) is mainly based on the approximate system error equation (Equation (11)), in order to obtain a more accurate identification, a new iterative algorithm, based on applying the Newton–Raphson procedure to Equation (5), is proposed:

(12) $$\begin{array}{ll} v\Omega_{k}\left(\omega_{i}\right) & =v\Omega_{k-1}\left(\omega_{i}\right)-{\left(\frac{\partial e\left(\omega_{i},v\Omega_{k-1}\left(\omega_{i}\right)\right)}{\partial v\Omega }\right)}^{-1}\cdot e\left(\omega_{i},v\Omega_{k-1}\left(\omega_{i}\right)\right)=\Theta \left(v\Omega_{k-1}\left(\omega_{i}\right)\right),\\ & \Theta \left(v\Omega \right)=v\Omega -{\left(\frac{\partial e\left(\omega_{i},v\Omega \right)}{\partial v\Omega }\right)}^{-1}\cdot e\left(\omega_{i},v\Omega \right),\\ & \frac{\partial e\left(\omega_{i},v\Omega \left(\omega_{i}\right)\right)}{\partial v\Omega }=\left[\begin{array}{ccc} \frac{\partial e_{1}}{\partial v\Omega_{1}} & \frac{\partial e_{1}}{\partial v\Omega_{2}} & \frac{\partial e_{1}}{\partial v\Omega_{2}}\\ \ldots & \ldots & \ldots \\ \frac{\partial e_{N_{m}}}{\partial v\Omega_{1}} & \frac{\partial e_{N_{m}}}{\partial v\Omega_{2}} & \frac{\partial e_{N_{m}}}{\partial v\Omega_{3}} \end{array}\right],\\ & \frac{\partial e_{s}}{\partial v\Omega_{r}}=-{\left(d\left(\omega_{i},1\right)\cdot \frac{\partial D^{-1}\left(v\Omega \left(\omega_{i}\right),\omega_{i}\right)}{\partial v\Omega_{r}}\cdot N\right)}_{S},\;\;s=1\ldots N_{m},\;\;r=1\ldots 3\\ & {\left(\frac{\partial D^{-1}\left(v\Omega \left(\omega_{i}\right),\omega_{i}\right)}{\partial v\Omega_{r}}\right)}_{s}=\\ & \;=-{\left(D^{-1}\left(v\Omega \left(\omega_{i}\right),\omega_{i}\right)\right)}_{s}\cdot {\left(\frac{\partial D\left(v\Omega \left(\omega_{i}\right),\omega_{i}\right)}{\partial v\Omega_{r}}\right)}_{s}\cdot {\left(D^{-1}\left(v\Omega \left(\omega_{i}\right),\omega_{i}\right)\right)}_{s}, \end{array}$$

where ∂**D**/∂*ν*Ω*_r_* is detailed in Equation (A16).

The end condition of the iterative procedure in Equation (12) is derived from Equation (10) by replacing $\tilde{e}\left(\omega_{i},v\Omega \left(\omega_{i}\right)\right)$ (Equation (10)) with $e\left(\omega_{i},v\Omega \left(\omega_{i}\right)\right)$ (Equation (5)). By using the end value obtained from the iterative procedure in Equation (9) as the initial input for this algorithm, the convergence is typically achieved within a few iterations, so that accurate **vΩ**(*ω_i_*) estimated values can be obtained. It is important to note that if, at the *k*-th iteration step, the **vΩ***_k_*(*ω_i_*) value obtained from Equation (12) does not satisfy Equation (3) conditions, the previously cited algorithm [30] can be applied to obtain **vΩ***_k_*(*ω_i_*) values, being consistent with the Equation (3) requirements.

**Figure 2:**
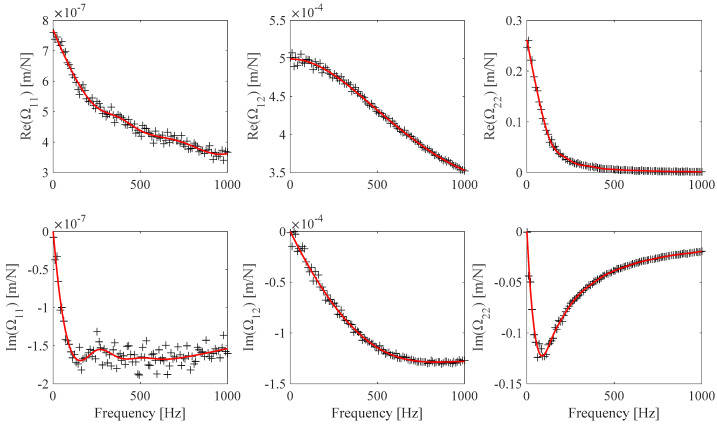
Identified **Ω** values (black) and B-spline fits with *n_cp_* optimal number of control points (red) (Ω_11_: *n_cp_* = 9, Ω_12_: *n_cp_* = 5, *n_cp_* Ω_22_: *n_cp_* = 15).

A smooth analytical **Ω**(*ω*) model can be obtained by fitting **vΩ**(*ω_i_*), *i* = 1…*Nω*, using 4th order polynomial B-spline functions, so that the contribution of the model and of the experimental noise is reduced. The resulting *n_cp_* optimal number of control points and the related optimal non-uniform weight vector of the B-spline curve fit were determined by means of the algorithm detailed in [33]. As a practical example, Figure 2 presents the discrete estimates of **Ω** along with their corresponding continuous B-spline fit curves.

## 3. Application Examples

### 3.1. Numerically Simulated Measurements

**Figure 3:**
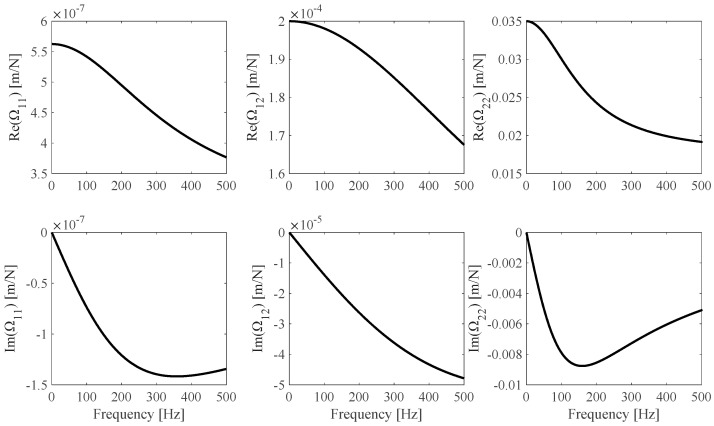
F1 **Ω** theoretical model.

The proposed identification procedure is validated by means of some numerical application examples. Numerically simulated measurements *h_s_*(*ω_i_*), *s* = 1…*N_m_* and *i* = 1…*N*_ω_ are obtained from Equation (4), where *N_m_* = 3 different specimens (BS1, Table 1) made of the same material (Im(*E*) = 0 = Im(*G*), *∂E*/*∂*ω = 0 = *∂G*/*∂ω*) are considered and *N*_ω_ = 500 linearly distributed frequency values in the [*f_min_* = 0.1, *f_max_* = 500] Hz range are considered as well. The F1 **Ω**(*ω*) theoretical model, illustrated in Figure 3, is used to model the frame.

**Figure 4:**
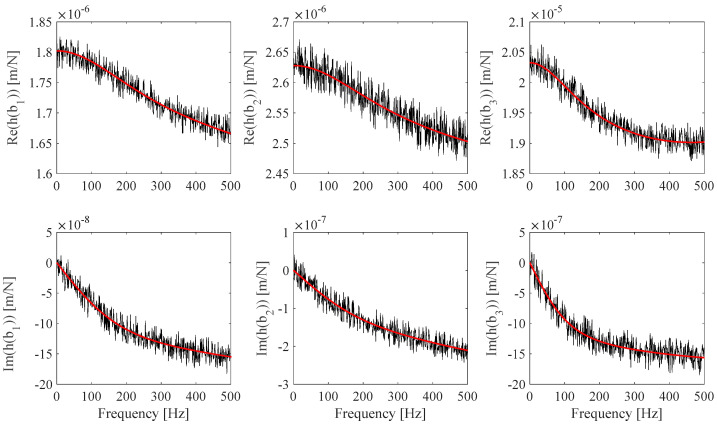
Numerically simulated measurements: *h_s_* (red) and ${\hat{h}}_{s}$ with S/N = 70 dB (black).

To test the robustness of the identification procedure, **Ω**(*ω*) is first estimated by assuming noiseless *h_s_*(*ω_i_*) simulated measurements, and then by taking into account ${\hat{h}}_{s}$(*ω_i_*) noisy simulated measurements, according to the following formula:

(13) $$\begin{array}{ll} {\hat{h}}_{s}\left(\omega_{i}\right)= & h_{s}\left(\omega_{i}\right)\cdot \left(1+10^{-\frac{S/N}{20}}\cdot \left(a\cdot e^{j\cdot b}\right)\right),\;\\ & a=rand(-1,1),\;\;b=rand(-\pi ,\pi ). \end{array}$$

where S/N is the assumed signal-to-noise ratio, *a* is a random variable between −1 and 1, and *b* is a random variable between −*π* and *π*. Figure 4 shows *h_s_*(*ω_i_*) and ${\hat{h}}_{s}$(*ω_i_*) with S/N = 70 dB simulated measurements plots.

**Figure 5:**
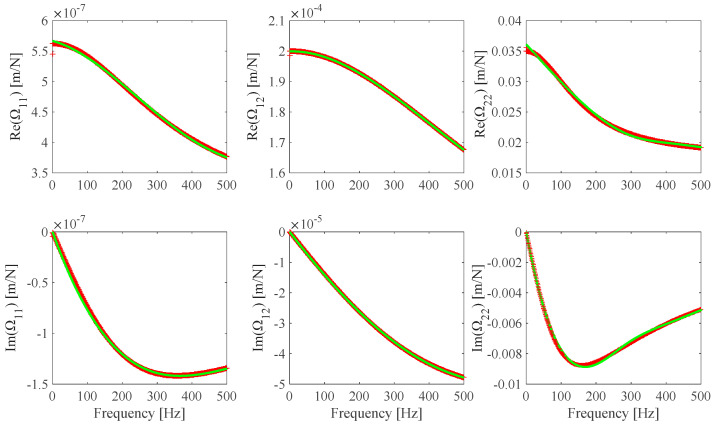
F1 **Ω** identified discrete values (red) and B-spline fits (Ω_11_: *n_cp_* = 6, Ω_12_: *n_cp_* = 6, *n_cp_* Ω_22_: *n_cp_* = 8) (green) from *h_s_* numerically simulated measurements with no added noise.

**Figure 6:**
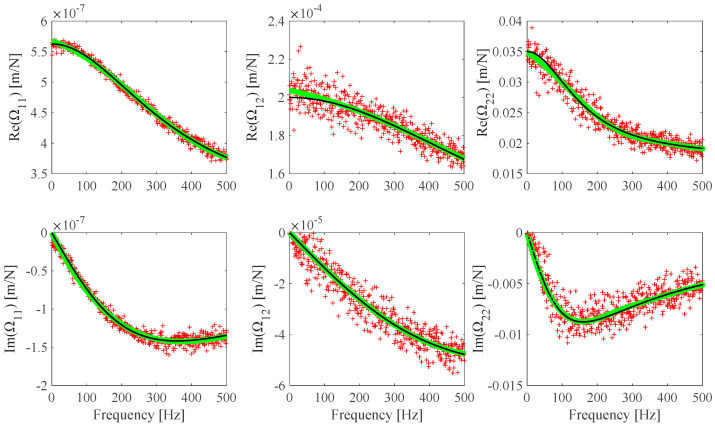
F1 **Ω** theoretical model (black), F1 **Ω** discrete identified values (red), and B-spline fits (Ω_11_: *n_cp_* = 6, Ω_12_: *n_cp_* = 6, *n_cp_* Ω_22_: *n_cp_* = 9) (green) from $\hat{h}$ numerically simulated measurements with S/N = 100 dB added noise.

**Figure 7:**
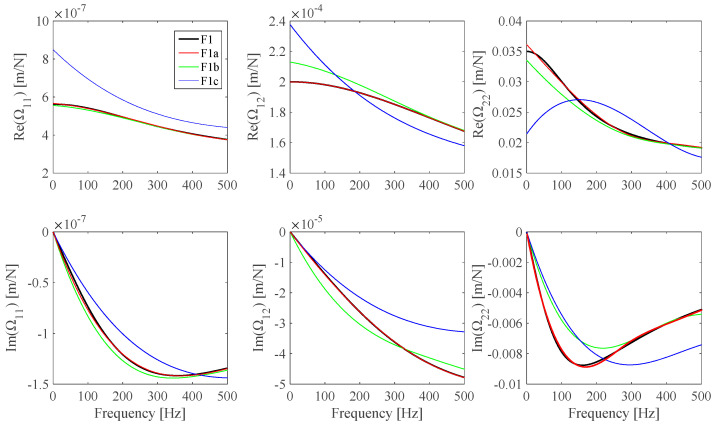
F1 **Ω**(*ω*) theoretical model (*F1t*, black) and B-spline fits from noiseless virtual measurements (*F1a*, red), S/N = 70 dB virtual measurements (*F1b*, green), and S/N = 40 dB virtual measurements (*F1c*, blue).

The **Ω**(*ω*) identification results obtained from noiseless measurements are shown in Figure 5, where the theoretical **Ω**(*ω*) plots, the identified **Ω**(*ω_i_*) discrete values, and the Ω_11_, Ω_12_, and Ω_22_ optimal B-spline fits are presented. It can be found that the identified **Ω**(*ω*) B-spline fitted model practically coincides with the theoretical model. **Ω**(*ω*) identification results from noisy measurements (S/N = 100 dB) are shown in Figure 6. Some **Ω**(*ω*) identification results from different noisy measurements associated with some S/N values are evaluated and shown in Figure 7. The identification error *Err*(Ω*_r,ℓ_*) related to the identified ${\left(\Omega_{r,𝓁}\left(\omega \right)\right)}_{id};\;\;r,𝓁=1,2$ optimal B-spline fits with respect to the ${\left(\Omega_{r,𝓁}\left(\omega \right)\right)}_{t};\;\;r,𝓁=1,2$ theoretical model is evaluated according to Equation (14).

**Figure 8:**
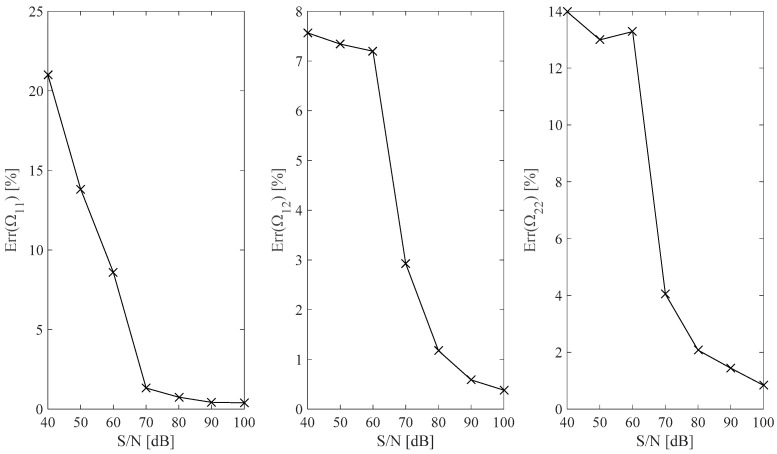
Relative error associated with Ω_11_, Ω_12_, and Ω_22_ identified B-spline fits with respect to the assumed measurement S/N ratio.

Optimal ${\left(\Omega_{r,𝓁}\left(\omega \right)\right)}_{id};\;\;r,𝓁=1,2$ B-spline fits are obtained by assuming different S/N values in the [40, 100] dB range and results are plotted in Figure 8.

(14) $$\;Err\left({\left(\Omega_{r,𝓁}\right)}_{id}\right)=\frac{1}{2\cdot \pi \cdot \left(f_{\max}-f_{\min}\right)}\cdot \int_{2\cdot \pi \cdot f_{\min}}^{2\cdot \pi \cdot f_{\max}}\left|1-\frac{{\left(\Omega_{r,𝓁}\left(\omega \right)\right)}_{id}}{{\left(\Omega_{r,𝓁}\left(\omega \right)\right)}_{t}}\right|d\omega \;;\;\;\;\;r,𝓁=1,2.$$

The results plotted in Figure 8 show that the identification error is generally low and monotonically tends to a negligible value by increasing the measurement S/N value assumption.

To show the contribution of accurate **Ω** frame identification on material identification estimates, a set of *h_s_*(*ω*) virtual measurements is numerically computed from the F1 **Ω**(*ω*) theoretical frame model and from the BS2 set of beam specimens reported in Table 2. BS2 beam specimens are made of the same material, according to a Standard Linear Solid (SLS) model [13,34] whose parameters are also reported in Table 2, making it possible to obtain *E*(*ω*) and *G*(*ω*) material properties by means of Equation (15).

(15) $$\frac{1}{E\left(\omega \right)}=\sum_{k=1}^{N_{E}}\frac{1}{E_{k}+j\cdot \omega \cdot \beta_{k}},\;\;\;\frac{1}{G\left(\omega \right)}=\sum_{k=1}^{N_{G}}\frac{1}{G_{k}+j\cdot \omega \cdot \eta_{k}}.$$

Since two unknown material parameters, formally varying with respect to frequency, need to be identified, *N_m_* ≥ 2 material specimens must be measured to obtain a consistent set of equations for the identification task. The geometric and material properties of the BS2 set are provided in Table 2, with *N_m_* = 3. A set of *h_s_*(*ω*) virtual measurements is numerically computed in the [*f_min_* = 0.1, *f_max_* = 500] Hz range, and *Nω* = 500 linearly distributed frequency values are assumed.

**Figure 9:**
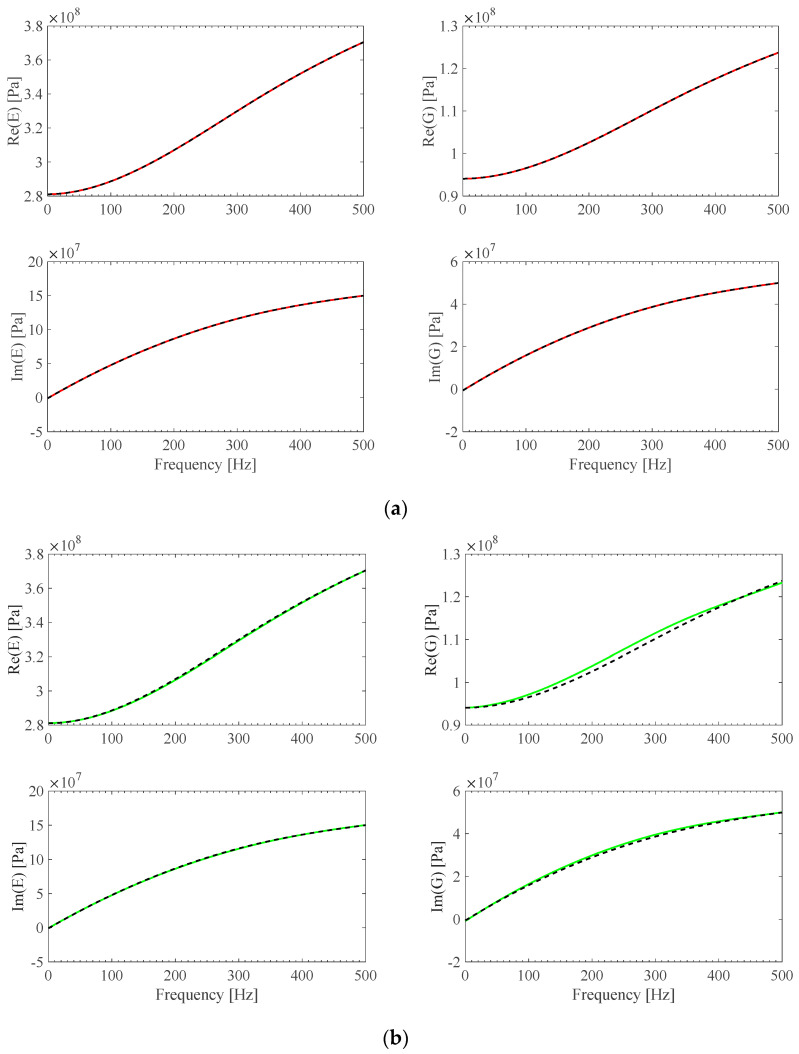

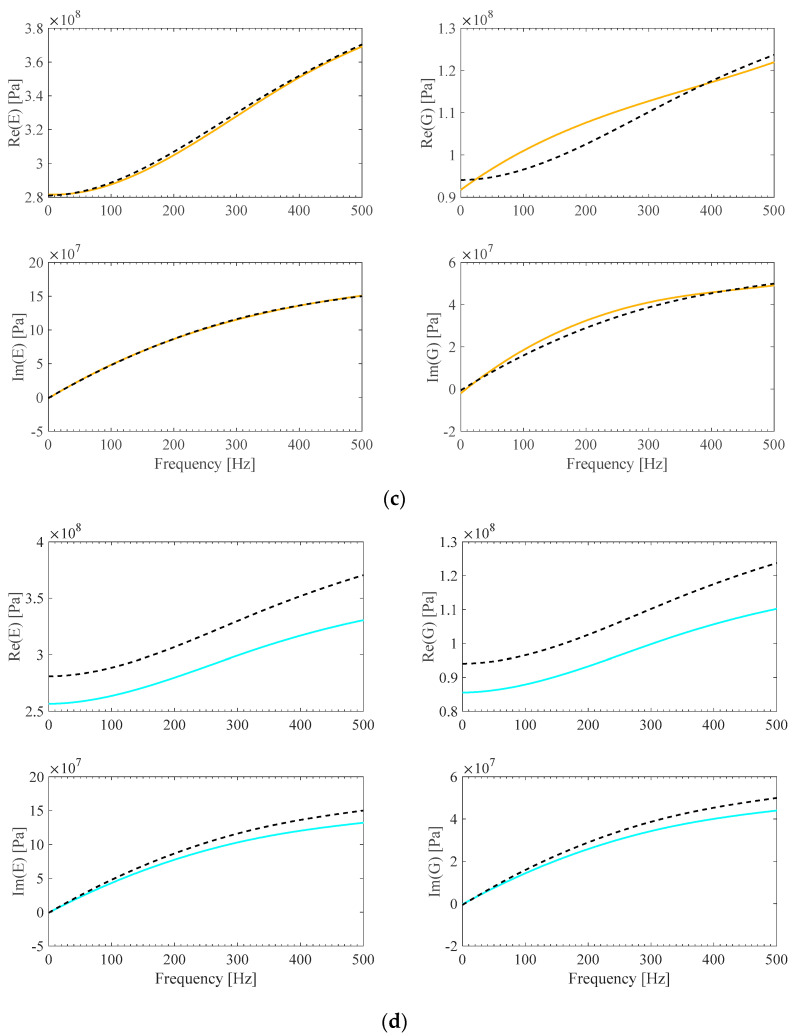
(**a**) *E*(*ω*) and G(*ω*) reference theoretical values (black) and *E*(*ω*) and G(*ω*) identified B-spline fits obtained from *F1a* **Ω** (red). (**b**) *E*(*ω*) and G(*ω*) reference theoretical values (black) and *E*(*ω*) and G(*ω*) identified B-spline fits obtained from *F1b* **Ω** (green). (**c**) *E*(*ω*) and G(*ω*) reference theoretical values (black) and *E*(*ω*) and G(*ω*) identified B-spline fits obtained from *F1c* **Ω** (orange). (**d**) *E*(*ω*) and G(*ω*) reference theoretical values (black) and *E*(*ω*) and G(*ω*) identified B-spline fits obtained from ideal **Ω** = **0** assumption (cyan).

A material identification procedure developed by these authors [13] was updated to identify the *E*(*ω*) and *G*(*ω*) parameters of the material of the BS2 specimens (Table 2). This was accomplished by considering the **Ω**(*ω*) F1 theoretical model first, and then the analytical **Ω**(*ω*) previously identified B-spline fits and the **Ω**(*ω*) = **0** ideal model as well. The results, shown in Figure 9a–d, illustrate the effect of the assumed frame model accuracy on material parameter identification.

**Figure 10:**
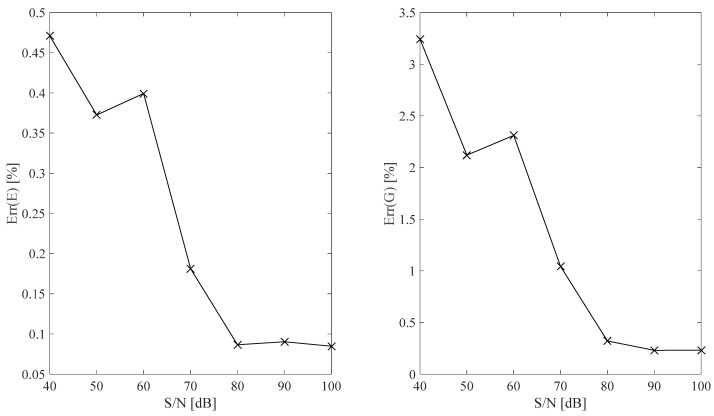
Relative error associated with *E* and *G* identified B-spline fits with respect to the assumed measurement S/N ratio used in F1 frame model identification.

The identification error *Err*(*E*, *G*) related to the identified ${\left(E\left(\omega \right)\right)}_{id},\;\;{\left(G\left(\omega \right)\right)}_{id}$ optimal B-spline fits with respect to the ${\left(E\left(\omega \right)\right)}_{t},\;\;{\left(G\left(\omega \right)\right)}_{t}$ theoretical model is evaluated according to Equation (16). Optimal ${\left(E\left(\omega \right)\right)}_{id},\;\;{\left(G\left(\omega \right)\right)}_{id}$ B-spline fits are obtained by assuming different S/N values in the [40, 100] dB range and results are plotted in Figure 10.

(16) $$\left\{\begin{array}{l} Err\left({\left(E\left(\omega \right)\right)}_{id}\right)=\frac{1}{2\cdot \pi \cdot \left(f_{\max}-f_{\min}\right)}\cdot \int_{2\cdot \pi \cdot f_{\min}}^{2\cdot \pi \cdot f_{\max}}\left|1-\frac{{\left(E\left(\omega \right)\right)}_{id}}{{\left(E\left(\omega \right)\right)}_{t}}\right|d\omega \;\\ Err\left({\left(G\left(\omega \right)\right)}_{id}\right)=\frac{1}{2\cdot \pi \cdot \left(f_{\max}-f_{\min}\right)}\cdot \int_{2\cdot \pi \cdot f_{\min}}^{2\cdot \pi f_{\max}}\left|1-\frac{{\left(G\left(\omega \right)\right)}_{id}}{{\left(G\left(\omega \right)\right)}_{t}}\right|d\omega \end{array}\right.$$

The results plotted in Figure 10 show that the identification error is typically low and tends toward a negligible value by increasing the measurement S/N value assumption.

**Figure 11:**
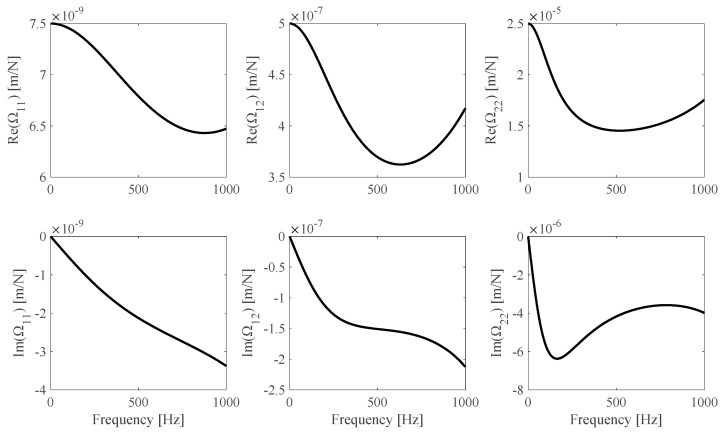
F2 **Ω** theoretical model.

An additional application example is reported. Numerically simulated measurements *h_s_*(*ω_i_*), *s* = 1… *N_m_*, and *i* = 1… *N_ω_*, are obtained from Equation (4), where *N_m_* = 3 different specimens (BS1, Table 1) made of the same material (Im(*E*) = 0 = Im(*G*), *∂E*/*∂ω* = 0 = *∂G*/*∂ω*) are considered and *N_ω_* = 1000 linearly distributed frequency values in the [*f_min_* = 0.1, *f_max_* = 1000] Hz range are considered as well. The F2 **Ω**(ω) theoretical model, illustrated in Figure 11, is used to model the frame.

**Figure 12:**
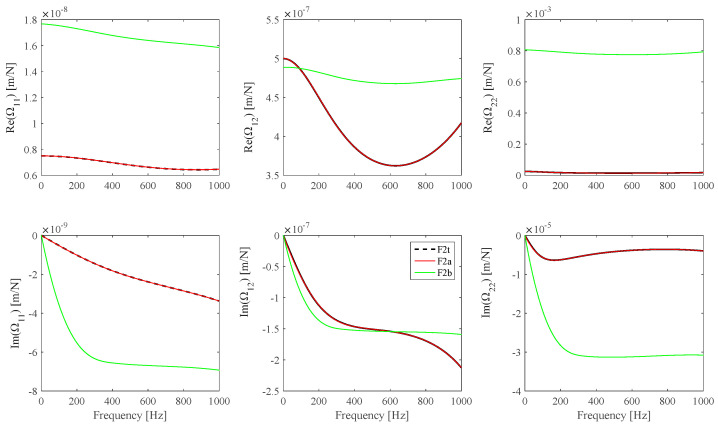
F2 **Ω** theoretical model (*F2t*, black) and B-spline fits from the current procedure (*F2a*, red) and from the previously proposed procedure (*F2b*, green).

Figure 12 presents the **Ω** identification results obtained by means of the identification procedure proposed in this work and the identification results obtained by means of an identification procedure previously proposed by these authors [28], which was based on Euler–Bernoulli beam theory assumptions. It can be observed that in this case, the previously proposed **Ω** identification procedure [28] gives inaccurate results. Different assumptions regarding the **Ω** frame model are expected to lead to variations in the identified material properties.

**Figure 13:**
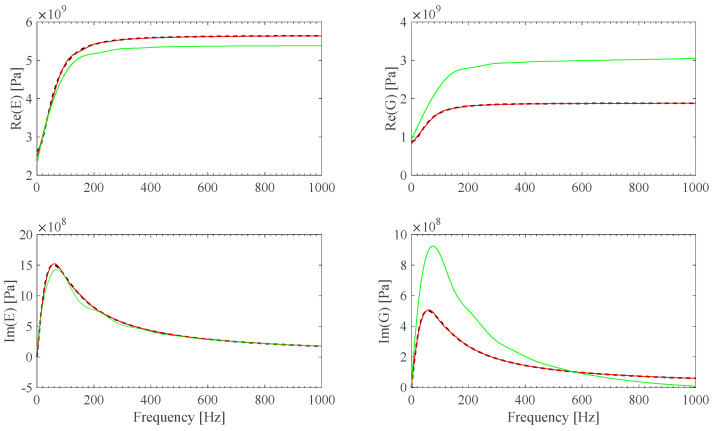
*E*(*ω*) and G(*ω*) reference values (black), and *E*(*ω*) and G(*ω*) identified B-spline fits obtained from *F2a* **Ω** (red) and *F2b* **Ω** (green).

As an example, a new set of virtual measurements is obtained using the F2 theoretical **Ω** frame model, the BS3 data set (reported in Table 3), and *N_m_* = 3 and *Nω* = 1000 linearly distributed frequency values in the [*f_min_* = 0.1, *f_max_* = 1000] Hz range. The BS3 material *E*(*ω*) and *G*(*ω*) continuous model fits are identified by means of the previously cited material identification procedure, taking into account the **Ω**(*ω*) F2 theoretical model first and then the analytical **Ω**(*ω*) previously identified B-spline fits by means of the procedure described in this work and the approximate procedure relying on the Euler beam model. Plot results are shown in Figure 13.

### 3.2. Experimental Measurements

**Figure 14:**
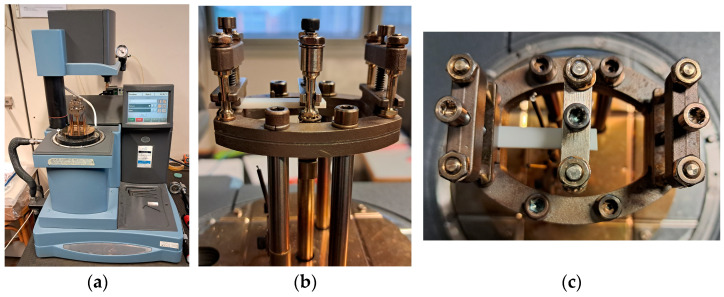
(**a**) DMA instrument; (**b**) EF1 clamping fixture lateral view; (**c**) EF1 clamping fixture upper view.

Forced vibration force and displacement measurements with sinusoidal excitation are conducted on a set of uniform homogeneous beams with rectangular cross section using a DMA Q800 apparatus (TA Instruments, New Castle, DE, USA) (Figure 14). Two different frame fixtures (EF1, EF2) are used to measure beam specimens of different geometry and are identified as well. Isothermal measurements, T = 25 °C, at *Nω* = 161 frequency values in the [*f_min_* = 0.01, *f_max_* = 150] Hz range are made on sets of harmonic steel specimens, whose related data are reported in Table 4 and Table 5. The frequency range is chosen by considering the instrument measurement range limits. Specimens made of harmonic steel material are used because the related material properties can be assumed to be known in the chosen frequency range. The lumped mass frame model parameter, common to both EF1 and EF2 frame configurations, is estimated through a preliminary specimen-free DMA input–output measurement campaign, considering ${\tilde{N}}_{\omega }$ measurements in the low frequency range:

(17) $$m=\frac{1}{{\tilde{N}}_{\omega }}\cdot \sum_{i=1}^{{\tilde{N}}_{\omega }}\frac{1}{\left|h\left(\omega_{i}\right)\right|\cdot \omega_{i}^{2}}=0.247\;kg$$

**Figure 15:**
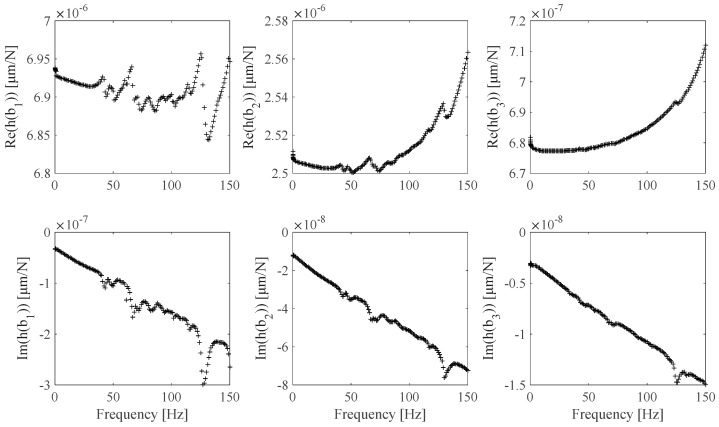
EF1 *h_s_* FRF experimental estimates related to **b**_1_, **b**_2_, and **b**_3_ specimens (Table 4).

**Figure 16:**
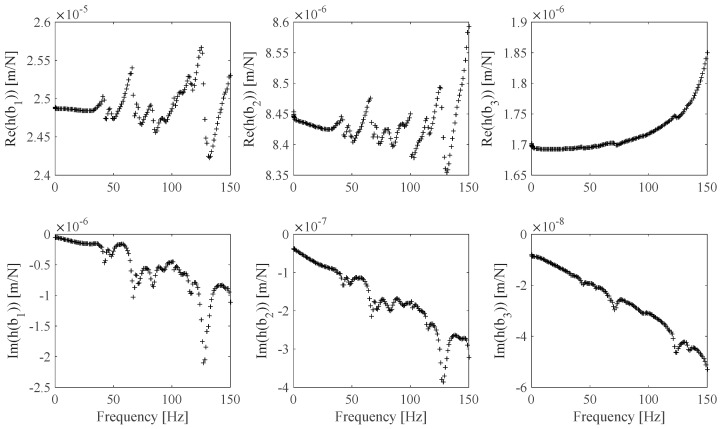
EF2 *h_s_* FRF experimental estimates related to **b**_1_, **b**_2_, and **b**_3_ specimens (Table 5).

**Figure 17:**
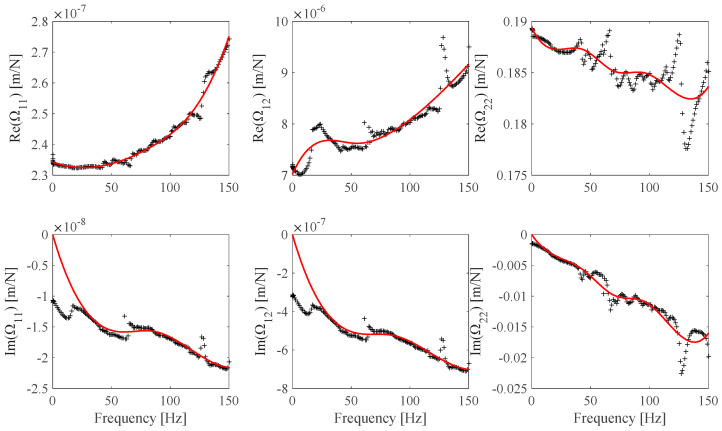
EF1 **Ω** identified discrete values (*EF1*, black) and B-spline fits (red) (Ω_11_: *n_cp_* = 7, Ω_12_: *n_cp_* = 7, *n_cp_* Ω_22_: *n_cp_* = 7).

**Figure 18:**
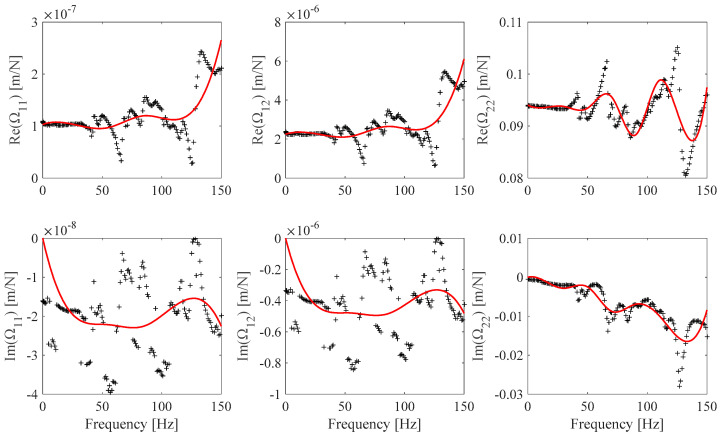
EF2 **Ω** identified discrete values (*EF1*, black) and B-spline fits (red) (Ω_11_: *n_cp_* = 6, Ω_12_: *n_cp_* = 6, *n_cp_* Ω_22_: *n_cp_* = 9).

The FRF *h_s_*(*ω*), *s* = 1 … *N_m_* estimates associated with the EF1 set (Table 4) and the EF2 set (Table 5) are shown in Figure 15 and Figure 16. The EF1, EF2 **Ω** frame identified discrete and continuous B-spline fit model results are shown in Figure 17 and Figure 18. Figure 17 shows the identified **Ω**(*ω*) values obtained by processing the FRF measurements shown in Figure 15 and the associated B-spline fit. Figure 18 shows the identified **Ω**(*ω*) values obtained by processing the FRF measurement shown in Figure 16 and the associated B-spline fit. It can be outlined that while the EF2 frame identified values appear to be affected by a higher measurement noise contribution than the EF1 frame identified values, the associated EF2 optimal B-spline fit shown in Figure 18 makes it possible to be effectively used in the proposed calibration procedure.

**Figure 19:**
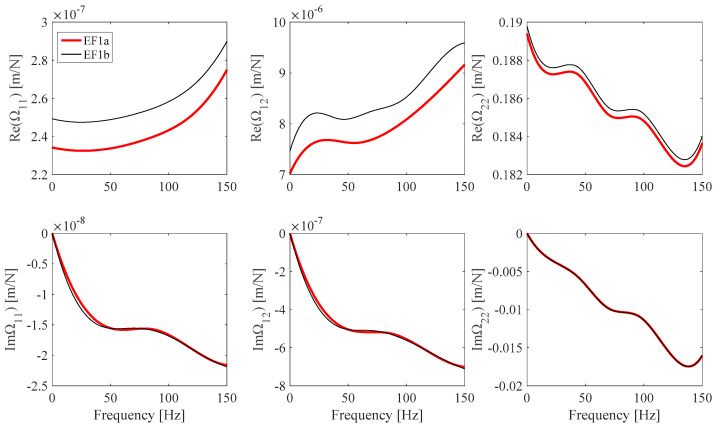
EF1 **Ω** identified B-spline fit (*EF1a,* red) and EF1 B-spline fit (Ω_11_: *n_cp_* = 7, Ω_12_: *n_cp_* = 7, *n_cp_* Ω_22_: *n_cp_* = 7) from the previously proposed procedure (*EF1b* black).

Figure 19 shows the EF1 frame **Ω**(*ω*) model obtained by means of the previously cited approximate frame calibration procedure based on Euler–Bernoulli beam theory. This model strongly differs from the one derived using the frame calibration procedure discussed in this work. It must be noted that since the reference beam specimens in Table 4 are made of harmonic steel, a stationary value of *E*(*ω*) = *E*(0) = 2.07·10^11^ GPa is assumed over the frequency measurement range. As a result, the contribution of the beam specimen to the Im(Ω_11_), Im(Ω_12_), and Im(Ω_22_) identified values is expected to be the same, regardless of the Euler or Timoshenko beam model assumptions. This result is clearly illustrated in the Im(Ω_11_), Im(Ω_12_), and Im(Ω_22_) plots shown in Figure 19. Moreover, since the Euler–Bernoulli beam model assumptions overestimate beam stiffness behavior with respect to real beam behavior, while the Timoshenko model assumptions more closely model real beam behavior, it is expected that the Re(Ω_11_), Re(Ω_12_), and Re(Ω_22_) values, identified using Euler–Bernoulli beam assumptions, overestimate the real frame compliance. The identified EF1 Re(Ω_11_), Re(Ω_12_), and Re(Ω_22_) values shown in Figure 19 agree with the expectations. Since the Re(Ω_22_) identified results mainly depend on *M*(0) and thus on the beam specimen length, the difference between the related identified values obtained by the different beam model assumptions is expected to be lower than the difference exhibited in the Re(Ω_11_) and Re(Ω_12_) results, and Figure 19 plots agree with this conclusion.

Discrete frame calibration results appear to be strongly influenced by measurement noise, but smooth continuous B-spline frame model fits, consistent with Equation (3) constraints and minimizing of noise contribution, can be easily obtained, as is shown in the results depicted in Figure 17 and Figure 18.

An application is proposed, consisting of *E*(*ω*) and G(*ω*) material model identification of two commercially available materials, polyvinyl chloride (PVC) and polytetrafluoroethylene (PTFE).

Isothermal measurements, T = 35 °C, at *Nω* = 161 frequency values in the [*f_min_* = 0.01, *f_max_* = 150] Hz range are made on the specimen sets, whose related data are reported in Table 6 and Table 7.

**Figure 20:**
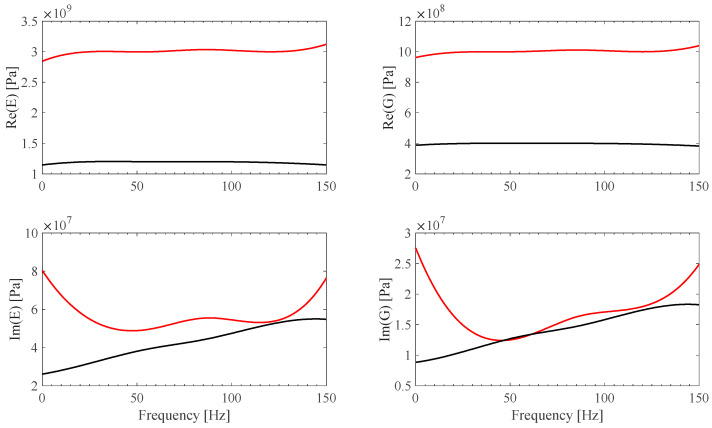
*E*(*ω*) and G(*ω*) identified B-spline fits from PVC measurements, using **Ω** calibration results for both EF1 and EF2 (red) and ideal **Ω**(*ω*) = **0** frame assumptions (black).

**Figure 21:**
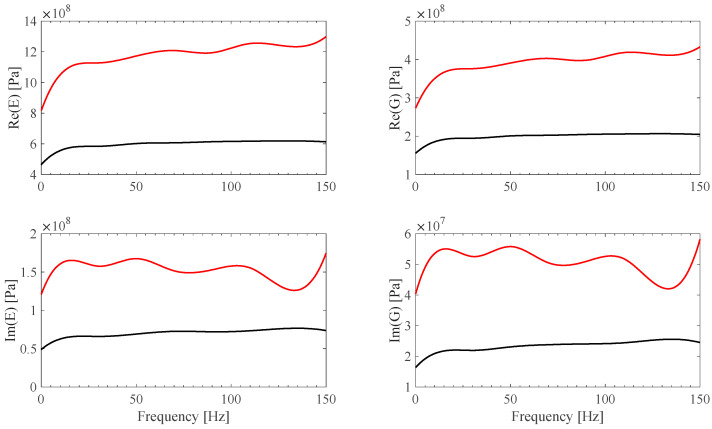
*E*(*ω*) and G(*ω*) identified B-spline fits from PTFE measurements, using **Ω** calibration results for both EF1 and EF2 (red) and ideal **Ω**(*ω*) = **0** frame assumptions (black).

*E*(*ω*) and G(*ω*) identified B-spline fit models are shown in Figure 20 and Figure 21. *E*(*ω*) and G(*ω*) material identified B-spline fit models, by assuming **Ω**(*ω*) = **0** ideal frame, are plotted in Figure 20 and Figure 21 as well. Both the PVC and PTFE material model identification results appear to be smooth, in the restricted measured experimental range, and consistent with expected value behavior [35,36]. It is also shown that results are strongly affected by the accuracy of the frame model, justifying the proposal of the sensor-based calibration procedure described in this work.

## 4. Conclusions

A novel procedure for the calibration of an experimental sensor-based test system for dynamical identification of a material model is presented. The proposed procedure relies on an inverse modeling approach, making it possible to obtain the dynamical model of the frame from within measurements performed on specimens mounted in the instrument frame, whose dynamical model is assumed by means of Timoshenko beam theory. The proposed technique is mainly based on a numerical, multi-step algebraic technique, generally not requiring the adoption of critical nonlinear optimization algorithms, which typically suffer from non-unique solution behavior.

The robustness of the procedure is investigated by means of some numerically generated virtual measurements, and consistent results are obtained even when high levels of random noise are added to the virtual measurements and taken into account. Identification results show that very accurate results can be obtained if simulated measurement S/N ≥ 60 dB. Such a value does not appear to be critical, since the precision of the measurement sensors and of the data acquisition and processing system to be used in a standard test system is expected to satisfy and overcome this requirement.

It must be indicated that the accuracy of the proposed calibration procedure is highly dependent on the selection of the reference beam specimen set to be tested. As a matter of fact, the condition number of the **BB** and ∂**e**/∂**vΩ** matrices (Equations (8) and (12)) strictly depends on it. A numerically ill-conditioned calibration procedure results if the condition number of the **BB** and ∂**e**/∂**vΩ** matrices exceeds a tolerance threshold, whose value depends on the computational hardware used for the task. For example, it is observed that increasing the difference between the length of each reference beam specimen a better-conditioned identification procedure results. It must be indicated that any critical choice in terms of the reference specimen set can be investigated in advance by means of a simple numerical simulation check, since **BB** and ∂**e**/∂**vΩ** matrices only depend on the reference beam specimen set geometrical and known material parameters and do not depend on measurements, so that any user can be warned in advance, if needed, before proceeding to measure the beam specimens. Moreover, by using such a simple simulation tool, an optimal reference specimen set can be found and used as well.

It can also be noted that the calibration technique is a multi-step procedure whose first step is related to the identification of the multi-dof frame model at each measured frequency value, so the accuracy of the whole procedure does not depend, in principle, on the choice of the frequency measurement range. Nevertheless, the choice of the frequency measurement range is generally limited by the specific measurement test apparatus to be used for the identification of the material properties.

Some real experimental applications are proposed, showing that the accuracy of the material model identification can be strongly affected by taking into account the frame contribution and the accuracy of its related dynamical model.

## Figures and Tables

**Table 1:** BS1 specimen data.

*N_m_* = 3	b_1_	b_2_	b_3_
Length [m]	1·10^−2^	1.5·10^−2^	2·10^−2^
Width [m]	2·10^−3^	4·10^−3^	3·10^−3^
Thickness [m]	2·10^−3^	2·10^−3^	1·10^−3^
Material: harmonic steel			
Density [kg/m^3^]	7850		
*E* [Pa]	2.07·10^11^		
*G* [Pa]	7.961·10^10^		

**Table 2:** BS2 specimen data.

*N_m_* = 3	b_1_	b_2_	b_3_
Length [m]	1·10^−2^	1.5·10^−2^	2·10^−2^
Width [m]	3·10^−3^	4·10^−3^	8·10^−3^
Thickness [m]	2·10^−3^	5·10^−3^	8·10^−3^
Material: theoretical virtual SLS model			
Density [kg/m^3^]	3100		
*N_E_*	*2*		
*E_k_* [Pa]	*E*_1_ = 5.04·10^8^, *E*_2_ = 6.37·10^8^		
*β_k_* [Pa·s]	*β*_1_ = 2.24·10^4^, *β*_2_ = 3.64·10^5^		
*N_G_*	*2*		
*G_k_* [Pa]	*G*_1_ = 1.68·10^8^, *G*_2_ = 2.12·10^8^		
*η_k_* [Pa·s]	*η*_1_ = 7.47·10^4^, *η*_2_ = 1.21·10^5^		

**Table 3:** BS3 specimen data.

*N_m_* = 3	b_1_	b_2_	b_3_
Length [m]	1·10^−2^	1.5·10^−2^	2·10^−2^
Width [m]	3·10^−3^	4·10^−3^	6·10^−3^
Thickness [m]	2·10^−3^	4·10^−3^	6·10^−3^
Material: theoretical virtual SLS model			
Density [kg/m^3^]	3100		
*N_E_*	*2*		
*E_k_* [Pa]	*E*_1_ = 5.65·10^9^, *E*_2_ = 4.95 10^9^		
*β_k_* [Pa·s]	*β*_1_ = 3.1·10^2^, *β*_2_ = 2.89·10^7^		
*N_G_*	*2*		
*G_k_* [Pa]	*G*_1_ = 1.89·10^9^, *G*_2_ = 1.65·10^9^		
*η_k_* [Pa·s]	*η*_1_ = 1.03·10^2^, *η*_2_ = 9.63·10^6^		

**Table 4:** EF1 **Ω**(*ω*) experimental frame identification specimens.

*N_m_* = 3	b_1_	b_2_	b_3_
Length [m]	1·10^−2^	1·10^−2^	1·10^−2^
Width [m]	1.27·10^−2^	1.27·10^−2^	2.99·10^−3^
Thickness [m]	0.5·10^−3^	0.75·10^−3^	2.99·10^−3^
Material: harmonic steel			
Density [kg/m^3^]	7850		
*E* [Pa]	2.07·10^11^		
*G* [Pa]	7.961·10^10^		

**Table 5:** EF2 **Ω**(*ω*) frame experimental identification specimens.

*N_m_* = 3	b_1_	b_2_	b_3_
Length [m]	1.75·10^−2^	1.75·10^−2^	1.75·10^−2^
Width [m]	1.27·10^−2^	1.27·10^−2^	2.99·10^−3^
Thickness [m]	0.5·10^−3^	0.75·10^−3^	2.99·10^−3^
Material: harmonic steel			
Density [kg/m^3^]	7850		
*E* [Pa]	2.07·10^11^		
*G* [Pa]	7.961·10^10^		

**Table 6:** PVC specimen data.

*N_m_* = 3	b_1_	b_2_	b_3_
Length [m]	1·10^−2^	1·10^−2^	1.75·10^−2^
Width [m]	8.52·10^−3^	1.084·10^−2^	8.52·10^−3^
Thickness [m]	6.01·10^−3^	4.07·10^−3^	6.01·10^−3^
Frame	EF1	EF1	EF2
Material: PVC			
Density [kg/m^3^]	1469		

**Table 7:** PTFE specimen data.

*N_m_* = 3	b_1_	b_2_	b_3_
Length [m]	1·10^−2^	1·10^−2^	1.75·10^−2^
Width [m]	1.073·10^−2^	1.088·10^−2^	1.073·10^−2^
Thickness [m]	5.05·10^−3^	3.91·10^−3^	5.05·10^−3^
Frame	EF1	EF1	EF2
Material: PTFE			
Density [kg/m^3^]	2145		

**Figure A1:**
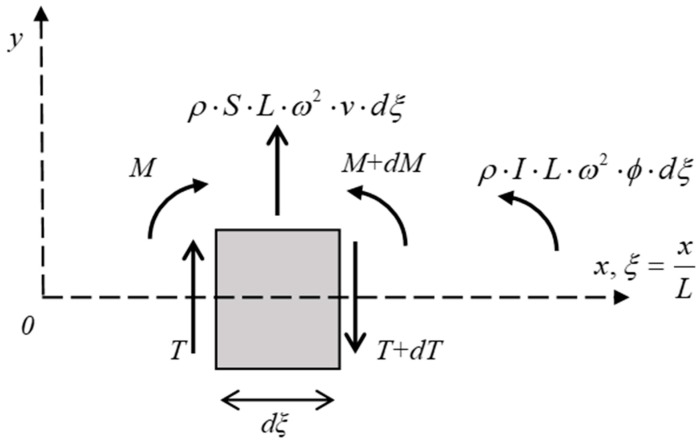
Dynamical actions on an infinitesimal length element of a beam.

**Figure A2:**
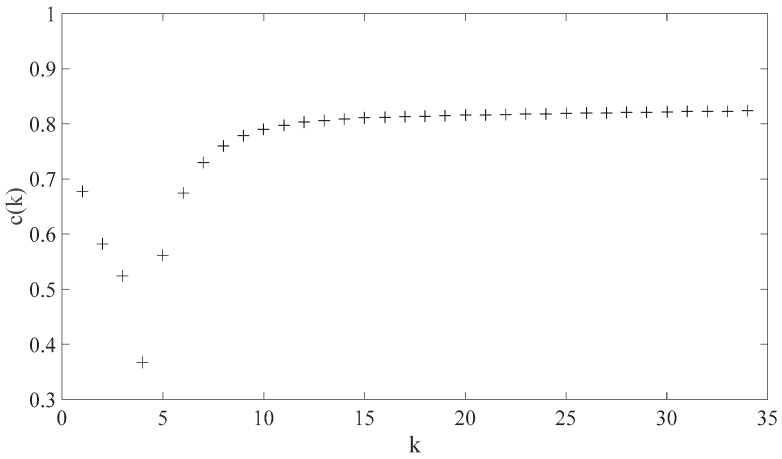
*c*(*k*) plot associated with a $\Phi \left(v\Omega \right)$ application example.

## Data Availability

Data related to simulated tests are shown in the paper tables. Experimental test data are available on request.

## Appendix A. Specimen–Instrument Frame Coupled System Model

Figure A1 shows the stress resultant components acting on an infinitesimal length element of a uniform, homogeneous, straight-beam specimen (Figure 1), taking into account both rotary inertia and shear contributions [29]. From the equilibrium conditions, the following results are obtained [29]:

(A1a) $$\frac{\partial T}{\partial \xi }=\rho \cdot S\cdot L\cdot \omega^{2}\cdot v,$$

(A1b) $$T=\frac{1}{L}\cdot \frac{\partial M}{\partial \xi }+\rho \cdot I\cdot \omega^{2}\cdot \phi ,$$

where *ρ*, *S*, refer to the material density and the beam section area, respectively, while *I* refers to the beam section moment. Moreover, the following results can be obtained:

(A2) $$u=-y\cdot \phi ;\;\;\varepsilon_{xx}=\frac{\partial u}{L\cdot \partial \xi }=-\frac{y}{L}\cdot \frac{\partial \phi }{\partial \xi };\;\;\gamma_{xy}=\frac{\partial u}{\partial y}+\frac{1}{L}\cdot \frac{\partial v}{\partial \xi }=-\phi +\frac{1}{L}\cdot \frac{\partial v}{\partial \xi };$$

where *ε_xx_* and *γ_xy_* are the axial and shear strain, respectively. From Equation (A2), material constitutive equations result:

(A3) $$\sigma_{xx}=E\cdot \varepsilon_{xx}=-\frac{E\cdot y}{L}\cdot \frac{\partial \phi }{\partial \xi };\;\;\;{\left(\tau_{xy}\right)}_{avg}=\kappa \cdot G\cdot \gamma_{xy}=\kappa \cdot G\cdot \left(\frac{1}{L}\cdot \frac{\partial v}{\partial \xi }-\phi \right),$$

where $\sigma_{xx},\;\;{\left(\tau_{xy}\right)}_{avg}$ are the axial stress and section averaged shear stress and *κ* refers to the Timoshenko shear coefficient [29]. The *κ* value can be approximated by means of the following formula proposed by Cowper [37] in the context of beam rectangular cross sections:

(A4) $$\kappa =\frac{10\cdot E}{2\cdot G+11\cdot E}.$$

The *M*, *T* beam section stress resultants are:

(A5a) $$M=-\int_{S}\sigma_{xx}\cdot y\cdot dS=\frac{E\cdot I}{L}\cdot \frac{\partial \phi }{\partial \xi },$$

(A5b) $$T=-\int_{S}\tau_{xy}\cdot dS=-\kappa \cdot G\cdot S\cdot \left(\frac{1}{L}\cdot \frac{\partial v}{\partial \xi }-\phi \right).$$

From Equations (A1a), (A1b) and (A5a):

(A6a) $$v=\frac{E\cdot I}{\omega^{2}\cdot \rho \cdot S\cdot L^{3}}\cdot \frac{\partial^{3}\phi }{\partial \xi^{3}}+\frac{I}{S\cdot L}\cdot \frac{\partial \phi }{\partial \xi }.$$

From Equations (A1b), (A5a) and (A5b):

(A6b) $$\phi =-\frac{1}{\omega^{2}\cdot \rho \cdot I-\kappa \cdot G\cdot S}\cdot \left(\frac{E\cdot I}{L^{2}}\cdot \frac{\partial^{2}\phi }{\partial \xi^{2}}+\frac{\kappa \cdot G\cdot S}{L}\cdot \frac{\partial v}{\partial \xi }\right).$$

From Equations (A6a) and (A6b):

(A7) $$\begin{array}{l} \frac{\partial^{4}\phi }{\partial \xi^{4}}+z_{1}^{2}\cdot \frac{\partial^{2}\phi }{\partial \xi^{2}}-\;z_{2}^{4}\cdot \phi =0,\;\;\;\frac{\partial^{4}v}{\partial \xi^{4}}+z_{1}^{2}\cdot \frac{\partial^{2}v}{\partial \xi^{2}}-\;z_{2}^{4}\cdot v=0,\\ \;\;z_{1}^{2}\left(\omega \right)=\frac{\omega^{2}\cdot \rho \cdot L^{2}}{E}\cdot \left(1+\frac{E}{\kappa \cdot G}\right),\;\;\;\;\;\;\;\;z_{2}^{4}\left(\omega \right)=\frac{\omega^{2}\cdot \rho \cdot S\cdot L^{4}}{E\cdot I}\cdot \left(1-\frac{\omega^{2}\cdot \rho \cdot I}{\kappa \cdot G\cdot S}\right). \end{array}$$

The solutions for homogeneous Equation (A7) are reported in Equation (A8).

(A8) $$v\left(\xi \right)=\alpha \cdot e^{\lambda \cdot \xi },\;\phi \left(\xi \right)=\beta \cdot e^{\lambda \cdot \xi };\;\;\lambda_{1,2}=\pm \theta ;\;\lambda_{3,4}=\pm \varphi ,$$

where:

(A9) $$\theta \left(\omega \right)=\sqrt{-\sqrt{\frac{z_{1}^{4}\left(\omega \right)}{4}+z_{2}^{4}\left(\omega \right)}-\frac{z_{1}^{2}\left(\omega \right)}{2}},\;\;\;\;\varphi \left(\omega \right)=\sqrt{\sqrt{\frac{z_{1}^{4}\left(\omega \right)}{4}+z_{2}^{4}\left(\omega \right)}-\frac{z_{1}^{2}\left(\omega \right)}{2}}.$$

The following results are obtained:

(A10) $$\begin{array}{l} \begin{array}{l} v\left(\omega ,\xi \right)=d\left(\omega ,\xi \right)\cdot \alpha ,\;\;\phi \left(\omega ,\xi \right)=d\left(\omega ,\xi \right)\cdot \beta .\\ \;\alpha ={\left[\begin{array}{cccc} \alpha_{1} & \alpha_{2} & \alpha_{3} & \alpha_{4} \end{array}\right]}^{T},\;\;\beta ={\left[\begin{array}{cccc} \beta_{1} & \beta_{2} & \beta_{3} & \beta_{4} \end{array}\right]}^{T}, \end{array}\\ \;d\left(\omega ,\xi \right)=\left[\begin{array}{cccc} \exp\left(\theta \left(\omega \right)\cdot \xi \right) & \exp\left(-\theta \left(\omega \right)\cdot \xi \right) & \exp\left(\varphi \left(\omega \right)\cdot \xi \right) & \exp\left(-\varphi \left(\omega \right)\cdot \xi \right) \end{array}\right] \end{array}.$$

From Equations (A1a) and (A5b):

(A11) $$\frac{\partial \phi }{\partial \xi }=\frac{\rho \cdot L\cdot \omega^{2}}{\kappa \cdot G}\cdot v+\frac{1}{L}\cdot \frac{\partial^{2}v}{\partial \xi^{2}}.$$

From Equations (A10) and (A11):

(A12) $$d\left(\omega ,\xi \right)\cdot \left[\begin{array}{c} \alpha_{1}\cdot \left(\frac{\theta^{2}}{L}+\frac{\rho \cdot \omega^{2}\cdot L}{G\cdot \kappa }\right)-\theta \cdot \beta_{1}\\ \alpha_{2}\cdot \left(\frac{\theta^{2}}{L}+\frac{\rho \cdot \omega^{2}\cdot L}{G\cdot \kappa }\right)+\theta \cdot \beta_{2}\\ \alpha_{3}\cdot \left(\frac{\varphi^{2}}{L}+\frac{\rho \cdot \omega^{2}\cdot L}{G\cdot \kappa }\right)-\varphi \cdot \beta_{3}\\ \alpha_{4}\cdot \left(\frac{\varphi^{2}}{L}+\frac{\rho \cdot \omega^{2}\cdot L}{G\cdot \kappa }\right)+\varphi \cdot \beta_{4} \end{array}\right]\;=0.$$

From Equation (A12), since **d**(*ω*,*ξ*) ≠ **0**:

(A13) $$\begin{array}{l} \beta ={\left[\begin{array}{cccc} \alpha_{1}\cdot \frac{\delta_{1}}{L} & -\alpha_{2}\cdot \frac{\delta_{1}}{L} & \alpha_{3}\cdot \frac{\delta_{2}}{L} & \alpha_{4}\cdot \frac{\delta_{2}}{L} \end{array}\right]}^{T}\\ \;\;\;\;\;\;\;\delta_{1}=\delta_{1}\left(\omega \right)=\theta +\frac{\rho \cdot \omega^{2}\cdot L^{2}}{G\cdot \kappa \cdot \theta },\;\;\;\;\;\;\;\delta_{2}=\delta_{2}\left(\omega \right)=\varphi +\frac{\rho \cdot \omega^{2}\cdot L^{2}}{G\cdot \kappa \cdot \varphi } \end{array}.$$

By imposing the boundary conditions depicted in Figure 1:

(A14) $$\left\{\begin{array}{l} v\left(\omega ,0\right)=-\Omega_{11}\cdot T\left(\omega ,0\right)+\Omega_{12}\cdot M\left(\omega ,0\right)\\ \phi \left(\omega ,0\right)=-\Omega_{12}\cdot T\left(\omega ,0\right)+\Omega_{22}\cdot M\left(\omega ,0\right)\\ \phi \left(\omega ,1\right)=0\\ T\left(\omega ,1\right)=-F-m\cdot \omega^{2}\cdot v\left(\omega ,1\right) \end{array}\right.$$

Equation (A14) in compact form is:

(A15) D⋅α=N⋅F.

**N** vector, **D**, *∂***D**/∂Ω*_rl_* matrices are:

(A16) $$\begin{array}{l} N={\left[\begin{array}{cccc} 0 & 0 & 0 & \frac{L}{S} \end{array}\right]}^{T},\;\;D=\left[\begin{array}{cccc} D_{11} & D_{12} & D_{13} & D_{14}\\ D_{21} & D_{22} & D_{23} & D_{24}\\ D_{31} & D_{32} & D_{33} & D_{34}\\ D_{41} & D_{42} & D_{43} & D_{44} \end{array}\right];\\ \;\;\;\;\;\;\;\;\;\;D_{11}=1-\Omega_{11}\cdot {ϒ}_{1}-\Omega_{12}\cdot {ϒ}_{3},\;\;D_{12}=1+\Omega_{11}\cdot {ϒ}_{1}-\Omega_{12}\cdot {ϒ}_{3},\\ \;\;\;\;\;\;\;\;\;D_{13}=1-\Omega_{11}\cdot {ϒ}_{2}-X_{12}\cdot {ϒ}_{4},\;\;D_{14}=1+\Omega_{11}\cdot {ϒ}_{2}-\Omega_{12}\cdot {ϒ}_{4},\\ \;\;\;\;\;\;\;\;\;D_{21}=\frac{\delta_{1}}{L}-\Omega_{12}\cdot {ϒ}_{1}-\Omega_{22}\cdot {ϒ}_{3},\;\;D_{22}=-\frac{\delta_{1}}{L}+\Omega_{12}\cdot {ϒ}_{1}-\Omega_{22}\cdot {ϒ}_{3},\\ \;\;\;\;\;\;\;\;\;D_{23}=\frac{\delta_{2}}{L}-\Omega_{12}\cdot {ϒ}_{2}-\Omega_{22}\cdot {ϒ}_{4},\;\;D_{24}=-\frac{\delta_{2}}{L}+\Omega_{12}\cdot {ϒ}_{2}-\Omega_{22}\cdot {ϒ}_{4},\\ \;\;\;\;\;\;\;\;\;D_{31}=\exp\left(\theta \right),\;\;\;\;\;D_{32}=\exp\left(-\theta \right),\;\;\;\;\;\;D_{33}=\frac{\delta_{2}}{\delta_{1}}\cdot \exp\left(\varphi \right),\;\;D_{34}=\frac{\delta_{2}}{\delta_{1}}\cdot \exp\left(-\varphi \right),\\ \;\;\;\;\;\;\;\;\;D_{41}=\kappa \cdot G\cdot \left(m\cdot \omega^{2}-\theta \right)\cdot \exp\left(\theta \right),\;\;\;D_{42}=\kappa \cdot G\cdot \left(m\cdot \omega^{2}+\theta \right)\cdot \exp\left(-\theta \right),\\ \;\;\;\;\;\;\;\;\;D_{43}=\kappa \cdot G\cdot \left(m\cdot \omega^{2}-\varphi \right)\cdot \exp\left(\varphi \right),\;\;\;D_{44}=\kappa \cdot G\cdot \left(m\cdot \omega^{2}+\varphi \right)\cdot \exp\left(-\varphi \right),\\ \;\;\;\;\;\;\;\;\;{ϒ}_{1}=\frac{S\cdot \left(\theta -\delta_{1}\right)\cdot \kappa \cdot G}{L};{ϒ}_{2}=\frac{S\cdot \left(\varphi -\beta \right)\cdot \kappa \cdot G}{L};\\ \;\;\;\;\;\;\;\;\;{ϒ}_{3}=\frac{I\cdot \theta \cdot \delta_{1}\cdot E}{L};\;\;\;\;{ϒ}_{4}=\frac{S\cdot \varphi \cdot \delta_{2}\cdot E}{L};\\ \;\;\;\;\;\;\;\;\;\frac{\partial D}{\partial \Omega_{11}}=\left[\begin{array}{cccc} -{ϒ}_{1} & {ϒ}_{1} & -{ϒ}_{2} & {ϒ}_{2}\\ 0 & 0 & 0 & 0\\ 0 & 0 & 0 & 0\\ 0 & 0 & 0 & 0 \end{array}\right];\;\;\;\;\;\frac{\partial D}{\partial \Omega_{12}}=\left[\begin{array}{cccc} -{ϒ}_{3} & -{ϒ}_{3} & -{ϒ}_{4} & -{ϒ}_{4}\\ -{ϒ}_{1} & {ϒ}_{1} & -{ϒ}_{2} & {ϒ}_{2}\\ 0 & 0 & 0 & 0\\ 0 & 0 & 0 & 0 \end{array}\right];\;\\ \;\;\;\;\;\;\;\;\;\;\;\;\;\;\;\;\frac{\partial D}{\partial \Omega_{22}}=\left[\begin{array}{llll} 0 & 0 & 0 & 0\\ -{ϒ}_{3} & -{ϒ}_{3} & -{ϒ}_{4} & -{ϒ}_{4}\\ 0 & 0 & 0 & 0\\ 0 & 0 & 0 & 0 \end{array}\right] \end{array}$$

From Equations (A10) and (A15), the FRF beam specimen *H*(*ω*) is:

(A17) $$H\left(\omega \right)=\frac{v\left(\omega ,\xi =1\right)}{F}=d\left(\omega ,1\right)\cdot D^{-1}\left(\omega \right)\cdot N.$$

The *H*(*ω*) approximate estimate can be obtained as follows. By neglecting the contribution of the distributed beam inertial actions with respect to the *F* excitation and the *m* lumped mass inertial contribution, the following result is obtained:

(A18) $$\left\{\begin{array}{l} M\left(\omega ,0\right)=L\cdot F+M\left(\omega ,1\right)\\ T\left(\omega ,0\right)=-F \end{array}\right.$$

Approximate boundary conditions result as well:

(A19) $$\left\{\begin{array}{l} v\left(\omega ,0\right)≃\Omega_{11}\cdot F+\Omega_{12}\cdot \left(L\cdot F+M\left(\omega ,1\right)\right)\\ \phi \left(\omega ,0\right)≃\Omega_{12}\cdot F+\Omega_{22}\cdot \left(L\cdot F+M\left(\omega ,1\right)\right)\\ \phi \left(\omega ,1\right)=0\\ T\left(\omega ,1\right)=-F-m\cdot \omega^{2}\cdot v\left(\omega ,1\right) \end{array}\right.$$

Equation (A19) in compact form is:

(A20) $$\begin{array}{l} A\left(\omega ,v\Omega \left(\omega \right)\right)\cdot \alpha_{F}=B\cdot v\Omega +C,\\ \;\;\;\;\;\;\;\alpha_{F}=\frac{1}{F}\cdot \alpha ,\;\;\;\;\;\;\;\;C={\left[\begin{array}{cccc} 0 & 0 & 0 & -\frac{L}{\kappa \cdot G\left(\omega \right)\cdot S} \end{array}\right]}^{T},\;\;\;\;\;\;\;B=\left[\begin{array}{lll} 1 & L & 0\\ 0 & 1 & L\\ 0 & 0 & 0\\ 0 & 0 & 0 \end{array}\right],\\ \;\;\;\;\;\;\;v\Omega ={\left[\Omega_{11},\;\Omega_{12},\;\Omega_{22}\right]}^{T}. \end{array}$$

The **A** matrix elements are:

(A21) $$\begin{array}{l} A_{11}=1-\Omega_{12}\cdot \frac{\theta \cdot \delta_{1}\cdot E\cdot I}{L^{2}}\cdot \exp\left(\theta \right),\;\;\;\;\;A_{12}=1-\Omega_{12}\cdot \frac{\theta \cdot \delta_{1}\cdot E\cdot I}{L^{2}}\cdot \exp\left(-\theta \right),\\ A_{13}=1-\Omega_{12}\cdot \frac{\varphi \cdot \delta_{2}\cdot E\cdot I}{L^{2}}\cdot \exp\left(\varphi \right),\;\;\;\;\;A_{14}=1-\Omega_{12}\cdot \frac{\varphi \cdot \delta_{2}\cdot E\cdot I}{L^{2}}\cdot \exp\left(-\varphi \right),\\ A_{21}=\frac{\delta_{1}}{L}-\Omega_{22}\cdot \frac{\theta \cdot \delta_{1}\cdot E\cdot I}{L^{2}}\cdot \exp\left(\theta \right),\;\;A_{22}=-\frac{\delta_{1}}{L}-\Omega_{22}\cdot \frac{\theta \cdot \delta_{1}\cdot E\cdot I}{L^{2}}\cdot \exp\left(-\theta \right),\\ A_{23}=\frac{\delta_{2}}{L}-\Omega_{22}\cdot \frac{\varphi \cdot \delta_{2}\cdot E\cdot I}{L^{2}}\cdot \exp\left(\varphi \right),\;\;A_{24}=-\frac{\delta_{2}}{L}-\Omega_{22}\cdot \frac{\varphi \cdot \delta_{2}\cdot E\cdot I}{L^{2}}\cdot \exp\left(-\varphi \right),\\ A_{31}=\frac{\delta_{1}}{L}\cdot \exp\left(\theta \right),A_{32}=-\frac{\delta_{1}}{L}\cdot \exp\left(-\theta \right),\;\;A_{33}=\frac{\delta_{2}}{L}\cdot \exp\left(\varphi \right),\;\;A_{34}=-\frac{\delta_{2}}{L}\cdot \exp\left(-\varphi \right),\\ A_{41}=-\theta \cdot \exp\left(\theta \right),\;\;A_{42}=\theta \cdot \exp\left(-\theta \right),\;\;A_{43}=-\varphi \cdot \exp\left(\varphi \right),\;\;A_{44}=\varphi \cdot \exp\left(-\varphi \right). \end{array}$$

From Equations (A10) and (A20), the beam specimen’s approximate *H*(ω) estimate is:

(A22) $$H\left(\omega ,v\Omega \left(\omega \right)\right)≃d\left(\omega ,1\right)\cdot A^{-1}\left(\omega ,v\Omega \left(\omega \right)\right)\cdot \left(B\cdot v\Omega \left(\omega \right)+C\right).$$

## Appendix B. Convergence Test for the Iterative Procedures

A convergence test procedure for the iterative procedures described in Equation (8) (**vΩ***_k_*= **Φ**(**vΩ***_k_*_−1_)) and Equation (12) (**vΩ***_k_*= **Θ**(**vΩ***_k_*_−1_)) is shown. An iterative procedure $x_{k}=\Lambda \left(x_{k-1}\right)$ converges to a unique fixed point if it satisfies the requirements associated with a contraction mapping [32].

Function Ψ:Γ→Γ is a contraction mapping on a compact Γ domain if ∃ c≤1:

(A23) $$\left|\Psi \left(x\right)-\Psi \left(y\right)\right|\leq c\cdot \left|x-y\right|,\forall x,y\in \Gamma .$$

It follows that **Ψ** has a unique $\bar{x}$ fixed point, i.e., $\bar{x}=\Psi \left(\bar{x}\right)$ [32], and for any $x_{0}\in \Gamma$, the following iterative, convergent series result:

(A24) $$x_{k}=\Psi \left(x_{k-1}\right),\;\;\;\;\;k=0,1,....$$

From Equation (A23):

(A25) $$\begin{array}{l} \left|\Psi \left(x_{1}\right)-\Psi \left(x_{0}\right)\right|=\left|x_{2}-x_{1}\right|\leq c\cdot \left|x_{1}-x_{0}\right|\\ \left|\Psi \left(x_{2}\right)-\Psi \left(x_{1}\right)\right|=\left|x_{3}-x_{2}\right|\leq c\cdot \left|x_{2}-x_{1}\right|\leq c^{2}\cdot \left|x_{1}-x_{0}\right|\\ \ldots \\ \left|\Psi \left(x_{k}\right)-\Psi \left(x_{k-1}\right)\right|=\left|x_{k+1}-x_{k}\right|\leq c^{k}\cdot \left|x_{1}-x_{0}\right| \end{array},$$

so that the minimum *c* value at the *k*-th iteration satisfying Equation (A25) is:

(A26) $$c\left(k\right)={\left(\frac{\left|x_{k+1}-x_{k}\right|}{\left|x_{1}-x_{0}\right|}\right)}^{\frac{1}{k}}.$$

In order to verify if $x_{k}=\Lambda \left(x_{k-1}\right)$ converges to a unique $\bar{x}$ fixed point starting from $x_{0},\;\;c\left(k\right)\leq 1$ condition can be checked for *k* increasing integer values. As an example, Figure A2 shows the *c*(*k*) test plot obtained with respect to the iterative **vΩ***_k_*= **Φ**(**vΩ***_k_*_−1_) mapping referred to in Equation (11), **vΩ**_0_ = **0**, from a specific application case.
